# Application of Recombinant Human scFv Antibody as a Powerful Tool to Monitor Nitrogen Fixing Biofertilizer in Rice and Legume

**DOI:** 10.1128/Spectrum.02094-21

**Published:** 2021-12-15

**Authors:** K. K. Khaing, K. Rangnoi, H. Michlits, N. Boonkerd, N. Teaumroong, P. Tittabutr, M. Yamabhai

**Affiliations:** a Molecular Biotechnology Laboratory, School of Biotechnology, Institute of Agricultural Technology, Suranaree University of Technology, Nakhon Ratchasima, Thailand; b Department of Chemistry, Institute of Biochemistry, BOKU - University of Natural Resources and Life Sciences, Vienna, Austria; c Biofertilizer & Plant-Microbe Interaction Research Unit, School of Biotechnology, Institute of Agricultural Technology, Suranaree University of Technology, Nakhon Ratchasima, Thailand; d Department of Plant Pathology, Yezin Agricultural University, Yezin, Nay Pyi Taw, Myanmar; University of Minnesota

**Keywords:** scFv, phage display, antibody, *Bradyrhizobium*, endophyte, rice-legume, nitrogen fixation, biofertilizer, symbiosis

## Abstract

*Bradyrhizobium* is an endophytic bacterium under investigation as an efficient biofertilizer for sustainable legume-rice rotational cropping system. Monitoring and bio-imaging of this nitrogen fixing bacterium is essential for the study of plant-microbe evolution, soil microbiome, as well as quality control in organic farming. While phage display antibody technology has been widely used to generate recombinant antibody for myriad medical purposes, so far, this technology has been minimally applied in the agricultural sector. In this study, single-chain variable fragments (scFv) against two *Bradyrhizobium* strains SUTN9-2 (yiN92-1e10) and DOA9 (yiDOA9-162) were isolated from a human phage display antibody library. Specific binding of scFv was demonstrated by ELISA and confocal-immunofluorescence imaging techniques. *Bradyrhizobium* localization in both endophytic and bacteroid forms could be observed inside rice tissue and plant nodule, respectively. Moreover, successful application of the recombinant antibody for the evaluation of nodule occupancy was also demonstrated in comparison with standard GUS-staining method. The results of this study showed for the first time the potential use of human phage display scFv antibody for imaging and monitoring of *Bradyrhizobium* biofertilizer and thus could be further applied for point-of-detection of bacterial inoculum in the legume-rice rotational crop system.

**IMPORTANCE** Human scFv antibody generated from phage display technology was successfully used for the generation of specific recombinant antibodies: yiN92-1e10 and yiDOA9-162 for the detection of *Bradyrhizobium* strains SUTN9-2 and DOA9, respectively. These two recombinant scFv antibodies could be used for precise detection of the rhizobia both in symbiosis with legume and endophyte in rice tissue by ELISA and immunofluorescent staining, during legume-rice rotational cropping system in the field. This methodology can be further employed for the study of other plant-microbe interactions and monitoring of biofertilizer in diverse sustainable cropping systems as well as in precision agriculture.

## INTRODUCTION

Cereals, including rice, are the major energy sources for mankind. Each year, a large amount of chemical nitrogen fertilizers made by Haber-Borsch process are being used to promote cereal growth and productivity (1). However, chemical nitrogen fertilizers can create several environmental and societal problems, such as the production process requiring large amount of fossil fuel, leading to greenhouse effects and global warming. Moreover, excess nitrogen cause nitrogen pollution, resulting in algae blooms, which affects human and aquatic animals (1). Proper distribution and accessibility of chemical fertilizer in poor regions also pose a challenge. Biological nitrogen fixation (BNF) in the form of biofertilizer using diazotrophic bacteria is an alternative source of nitrogen for cereal production (2). This method is sustainable and more efficient than the chemical methods. However, rhizobium-plant interactions are highly complex, especially for nitrogen-fixing symbiosis with legumes because rhizobia are phylogenetically, metabolically, and genetically diverse (3). Among a diverse group of diazotrophs, *Bradyrhizobium* is a group of slow growing Gram-negative soil bacteria, many of which can fix nitrogen symbiotically with specific legumes by converting nitrogen gas into ammonia to be used as fertilizer in plants (2). This symbiosis becomes one of the most remarkable factors in agriculture, as it supplies large quantity of nitrogen to the world ecosphere (3). *Bradyrhizobium* can thrive in symbiotic and endophytic associations with leguminous and nonleguminous plants. *Bradyrhizobium* sp. strain SUTN9-2 is an endophytic bacterium in rice tissues that can fix nitrogen as well as produce plant hormone (indole acetic acid; IAA) and enzyme 1-aminocyclopropane-1-carboxylic acid (ACC) deaminase, which can reduce plant stress and support plant growth (4). Therefore, this bacterium can be applied as biofertilizer/biostimulant in rice-legume crop rotation system. Since symbiosis of rhizobium and plant host is highly specific (3), there is need to identify and select a superior strain to be used as rhizobium inoculants in a biofertilizer for specific crops (5). Monitoring of rhizobium inoculant after application in rice-legume cropping system is also important to ascertain its persistence and efficiency in plant growth promotion (4). Moreover, precise identification of particular diazotrophic bacteria in different plant hosts will also be useful for the study of plant-microbe interactions and evolution of symbiotic nitrogen-fixing bacteria as well as the control of nodulation and intracellular infection in plant host (3).

Currently, enzyme-linked immunosorbent assay (ELISA) is the most common immunological method for the identification and monitoring of rhizobia. Nonetheless, polyclonal antibody exhibited cross-reactivity with other rhizobial strains within the same species (6). This cross-reaction is a major concern for detection and monitoring of specific rhizobium by polyclonal antibodies. The detection of *Rhizobium trifolli* 162X95 with monoclonal antibody by indirect ELISA showed high specificity (7). However, the production of monoclonal antibody by traditional method is extremely laborious, time-consuming, and entails comparatively high production costs. Moreover, all of the assays have in common the use of antibodies raising in animals (8).

Recombinant antibodies have the potential to accompany or replace hybridoma technology for monoclonal antibody production because the long-term cost for the production of antibody would be lower and the antibody could be acquired for diverse biosensor formats, allowing easy access to broader range of end users (9). Phage display technology utilizes libraries of recombinant bacteriophages that expose the antibody on their surface, and it permits the isolation of recombinant antibodies with the desirable binding affinity against the antigen by an iterative selection procedure (10). The key advantage of this technology is the direct linkage between genotype and phenotype (11), allowing further engineering or large-scale production of recombinant antibodies form Escherichia coli or other appropriate expression hosts without the use of experimental animals (12). The technology acquired a Nobel Prize in chemistry in the year 2018 (13).

From numerous recombinant antibody formats, a single chain fragment variable (scFv) is greatly desired. An scFv molecule consists of a variable region of heavy chains and light chains that are linked together by a flexible peptide linker. The advantages of scFv molecules are their small size, stability, and the ability to be engineered and produced at a large scale (8). Phage antibody display technology has been successfully used for the isolation of specific peptides and monoclonal antibodies against various targets including various pathogens, viruses, parasites, and mycotoxins (14–17).

In the aspect of rhizobial research, phage-display of rabbit scFv antibody technology has been used for the specific detection of *Bradyrhizobium* sp. strain DOA9 (18). However, rabbit antibody requires animal immunization and sacrifice and in general, recombinant antibody from rabbit is difficult to express (19). A naive human phage display antibody library is an attractive alternative source of recombinant antibody against rhizobium because it is possible that human population have been exposed to several rhizobia in the environment (12). So far, the use of phage display human antibody library is mainly focused on therapeutic purposes. Generation of specific human recombinant scFv antibody against beneficial soil bacteria using phage display technique has never been reported. In this study, phage-display antibody technology has been used to produce specific human recombinant scFv antibodies against two *Bradyrhizobium* strains (i.e., DOA9 and SUTN9-2). Applications of the recombinant antibody for detection, bio-imaging, and monitoring of this beneficial rhizobium in symbiosis with leguminous (mung bean) host and endophytic associations with nonleguminous (rice) plant are demonstrated. The efficiency of this new method for determination of nodule occupancy is compared with the standard method that employed the tagged strain using β-glucuronidase (GUS) reporter system.

## RESULTS

### Affinity selection (biopanning) of specific scFv antibodies against *Bradyrhizobium* strains SUTN9-2 and DOA9 from a compact human phage display antibody library.

A simple biopanning procedure was performed against *Bradyrhizobium* strains SUTN9-2 and DOA9 using nonimmunized human Yamo I phage-displayed scFv antibody library (12), based on our previous method for the isolation of antibacterial scFv (17). A summary of biopanning results is listed in Table 1. After the first round of biopanning, 1.3 × 10^2^ clones against SUTN9-2 and 1.4 × 10^2^ clones against DOA9 were obtained. Then, 96 and 144 colonies against SUTN9-2 and DOA9, respectively, were manually picked and were subjected to monoclonal phage ELISA to identify bona fide binder. For DOA9, three positive virion clones showing twofold higher optical density (OD) values than negative control (1% bovine serum albumin [BSA]) were identified (Fig. S1A in the supplemental material). After that, the binding specificity of these three clones were confirmed by cross-reactivity checking with other *Bradyhizobium* and other bacteria (Fig. S2A in the supplemental material). While clone D5 cross-react with almost every other bacterium, clone A1/2 and D1/6 could bind to both DOA9 and SUTN1-12 but binding signal of clone D1/6 against SUTN1-12 was much lower than that of DOA9. None of these two clones showed cross-react with SUTN9-2. After that, clone A1/2 and D1/6 clones were produced as soluble scFv using nonsuppressor strain of E. coli (HB2151) (15). Out of two clones, only clone D1/6 could be produced and showed high signal by scFv ELISA (data not shown). This clone was then designated as yiDOA9-162 and sub-clone into pET-21d (+) expression system, which is more efficient for lab-scale production for further characterization (17).

**TABLE 1:** Summary of affinity selection results against *Bradyrhizobium* strains SUTN9-2 and DOA9

Affinity selection step	*Bradyrhizobium* strain SUTN9-2	*Bradyrhizobium* strain DOA9
Round of selection	1	1
Colonies obtained	1.3 × 10^2^	1.4 × 10^2^
Colonies picked up	96	144
Positive clones at monoclonal phage ELISA	2	3
No. of expressed soluble scFv	2/2	2/3
Positive clones at scFv ELISA expressed in HB2151 Escherichia coli	1/2	1/2
No. of different scFv clones	1 (yiN92-1e10)	1 (yiDOA9-162)

For SUTN9-2, two positive phage clones (OD value was two times higher than that of 1% BSA control) were obtained as indicated by phage ELISA (Fig. S1B). Afterwards, binding specificity of these two clones were confirmed by phage ELISA against different *Bradyrhizobium* strains (Fig. S2B). After these two clones were produced as soluble scFv from the nonsuppressor strain of E. coli (HB2151), we found that only 1 clone that exhibited high binding signal by scFv ELISA (data not shown). Therefore, this clone was designated as yiN92-1e10, and further expressed from E. coli using the pET-21d (+) expression system for further analysis.

**FIG 1:**
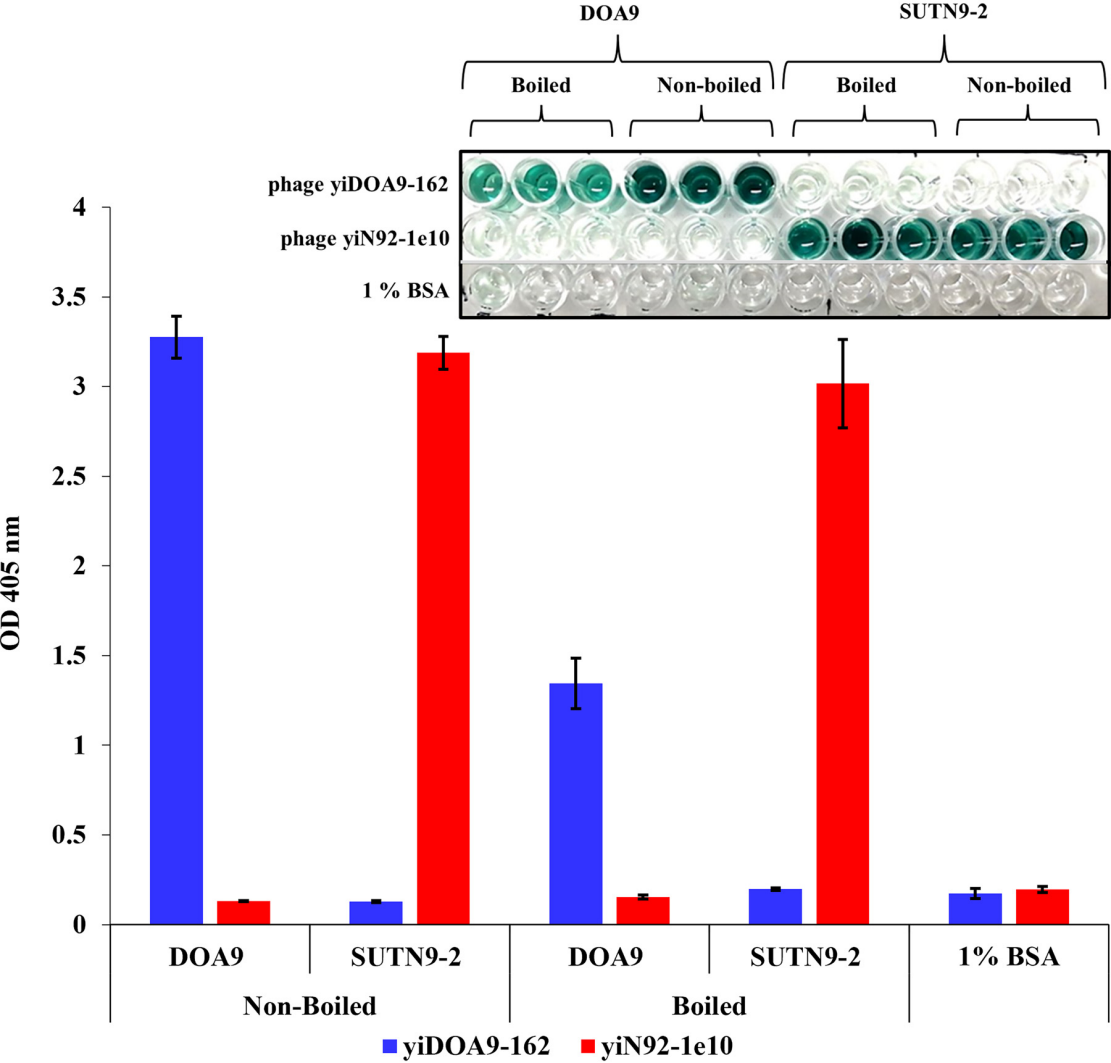
Specific binding of selected phage-displayed scFv clones. Phage ELISA result of the binding of scFv antibody clones yiDOA9-162 and yiN92-1e10 against strain DOA9 and SUTN9-2, respectively in pure culture (boiled and nonboiled cells). The average OD_405_ nm values and standard errors from triplicated wells are shown.

The binding of selected phage clones was confirmed by phage enzyme-linked immunosorbent assay (ELISA) against strains SUTN9-2 and DOA9 (boiled and nonboiled) cells. The two recombinant phage-displayed human scFv clones showed specific binding against their target strains (Fig. 1). These two scFv clones that interact specifically with *Bradyrhizobium* strain SUTN9-2 and DOA9 were designated as yiN92-1e10 and yiDOA9-162, respectively (patent application submitted). The amino acid sequences of these two scFv fragments are shown in Fig. S3A in the supplemental material. The three-dimensional structure prediction of these two clones is shown in Fig. S3B. The complementarity-determining region (CDR1, CDR2, and CDR3) of the VH and VL were indicated by PyMOL program. The VH and VL fragments of scFv yiN92-1e10 were derived from germ lines IGHV3-64*07 F and IGKV3-15*01 F (DPK21), which belong to subgroup VH3 and VK3, respectively. The VH and VL fragments of scFv yiDOA9-162 were derived from germ lines IGHV4-59*01 F (DP71) and IGKV3-20*01 F (DPK22), which belong to subgroup VH4 and VK3, respectively.

### Production of soluble scFv antibody fragments.

**FIG 2:**
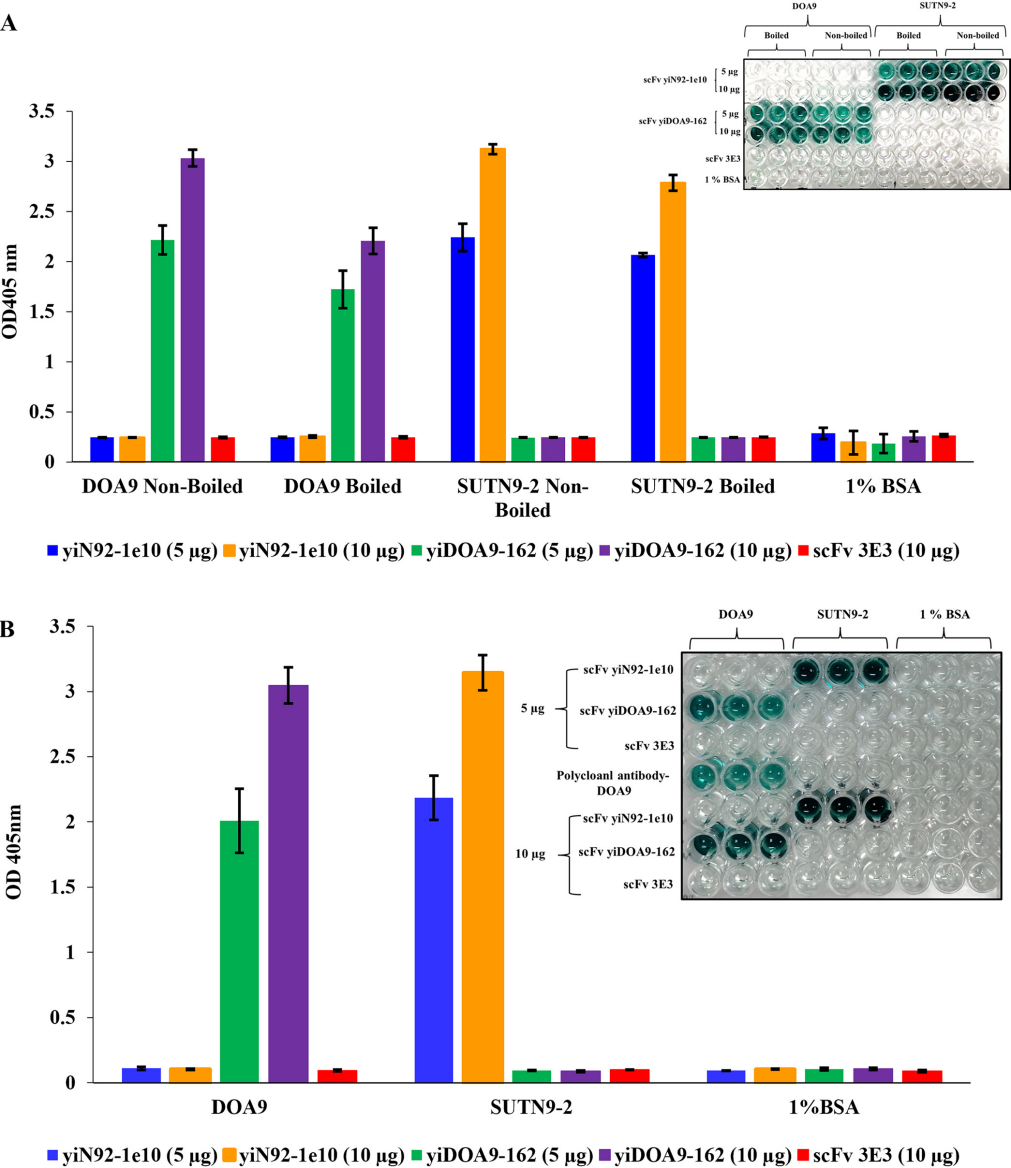
Binding property of free soluble scFv antibodies. ELISA results of soluble scFv antibodies (yiN92-1e10 and yiDOA9-162) against each target strain in planktonic form (A) and bacteroid form inside the nodules of *Bradyrhizobium* DOA9 and SUTN9-2 obtained from *A*. *americana* and *V*. *radiata*, respectively (B). Values are the mean of triplicate wells. Error bars show the standard deviation for each set of data.

To demonstrate the binding of free scFv antibody independent of the phage particle, the soluble form of scFv antibodies yiN92-1e10 and yiDOA9-162 against strains SUTN9-2 and DOA9, respectively, were produced by subcloning the scFv genes from phage display phagemid vector, pMod I, into pET21d (+) vector between NcoI and NotI sites and expressed in an E. coli host that promotes disulfide bond formation in cytosol (20). The recombinant scFv antibodies were expressed under the induction of isopropyl-β-d-thiogalactopyranoside (IPTG). After induction, cell pellets were collected and lysed by ultrasonic disruption. The supernatant containing recombinant antibody was first purified by one-step immobilized metal affinity chromatography. However, a large amount of contaminated co-eluting proteins was observed; therefore, the eluted fractions were purified again using the same column. The size of the purified scFv was approximately 30 kDa on SDS-PAGE as expected (Fig. S4). The binding property of purified scFv clones against strains SUTN9-2 and DOA9, both in the planktonic and bacteroid forms inside the nodule of *Vigna radiata* and *Aeschynomene americana*, respectively are illustrated in Fig. 2. These results demonstrated that free scFv antibodies retain specific binding activity against both forms of each target strain. The ELISA signals indicated that 5 μg/well of antibody could be used to detect 5 μg/well of total protein of target *Bradyrhizobium* cells, and 200 μl suspension of 5 nodules/ml of the bacteroid forms. The negative control 3E3 scFv antibody (21) showed no binding signal against either *Bradyrhizobium* strains SUTN9-2 or DOA9, in both pure culture (Fig. 2A) and bacteroid forms (Fig. 2B).

### Immunofluorescence staining of *Bradyrhizobium* strains in pure culture and nodule.

**FIG 3:**
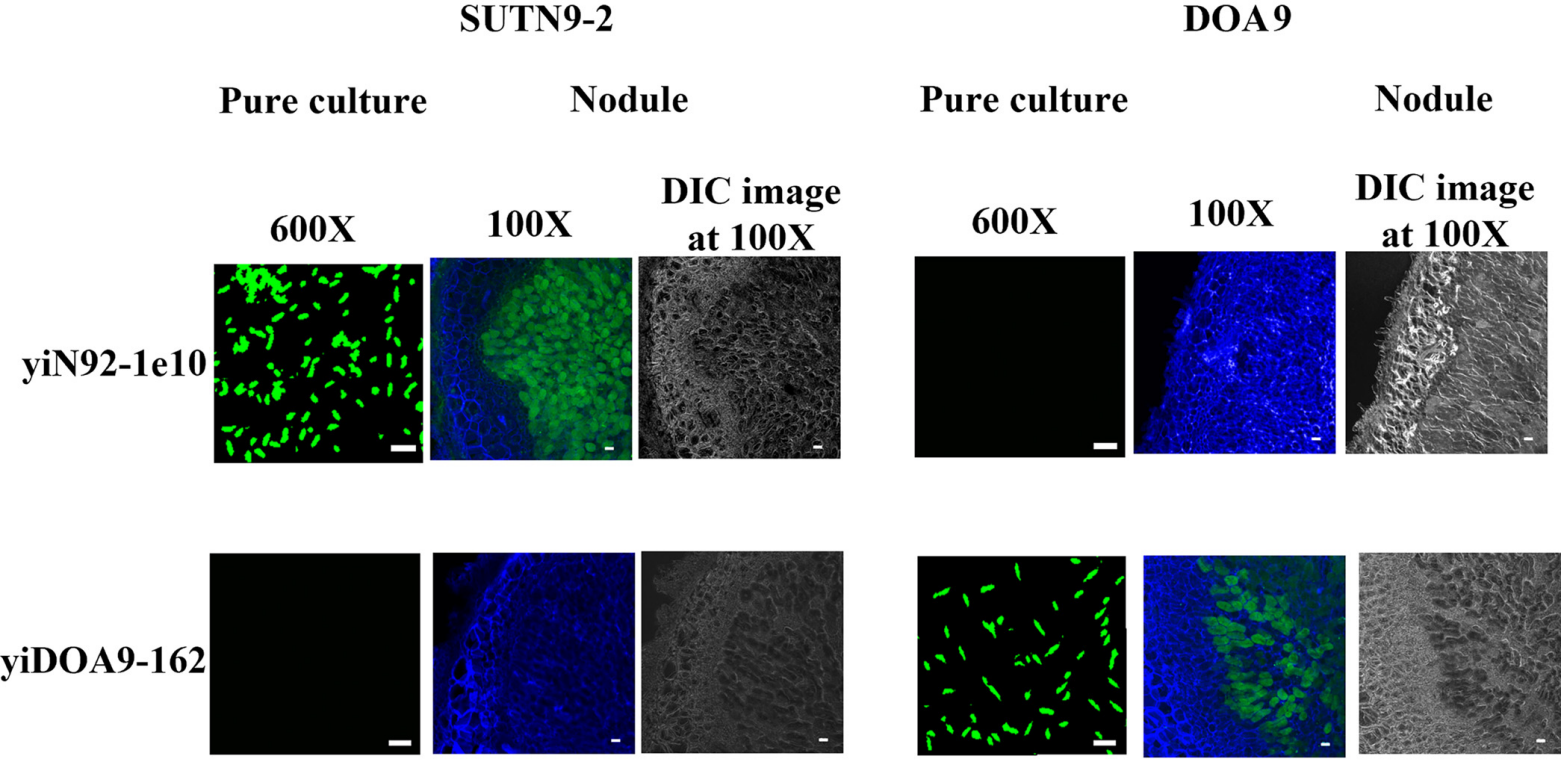
Confocal laser scanning micrographs of *Bradyrhizobium* strains SUTN9-2 and DOA9 prepared from pure culture and plant nodules. The bacterial samples were stained with two different scFv antibodies: yiN92-1e10 and yiDOA9-162. Green spot indicated the green-fluorescent staining of scFv antibody, using secondary antibody conjugated to anti-His-Dylight 488. Plant cell walls were stained with a blue fluorophore (Calcofluor white M2R) and emitted blue color. The bacteroides are shown as green spots inside a blue plant cavity. The nodule images observed by differential interference contrast (DIC) technique were also indicated. Scale bar is 10 μm at ×600 magnification, and 100 μm at ×100 magnification for pure culture and nodule samples, respectively.

Immunofluorescence analysis was performed to confirm the specific binding of the free soluble scFv and to visualize the morphology of the bacteroid form inside plant nodule directly under the microscope. The scFv clone yiDOA9-162 could bind specifically to strain DOA9 and scFv clone yiN92-1e10 exhibited specific binding to strain SUTN9-2 (Fig. 3). There was no cross-reactivity between the two scFv clones against strains DOA9 and SUTN9-2 in both pure culture and bacteroid forms. In comparison with the image obtained from bright field, the morphology of symbiotic nodules could be observed as green fluorescence bacteroid surrounded by blue plant cell wall from Calcofluor staining.

### Cross-reactivity checking of scFv antibody.

**FIG 4:**
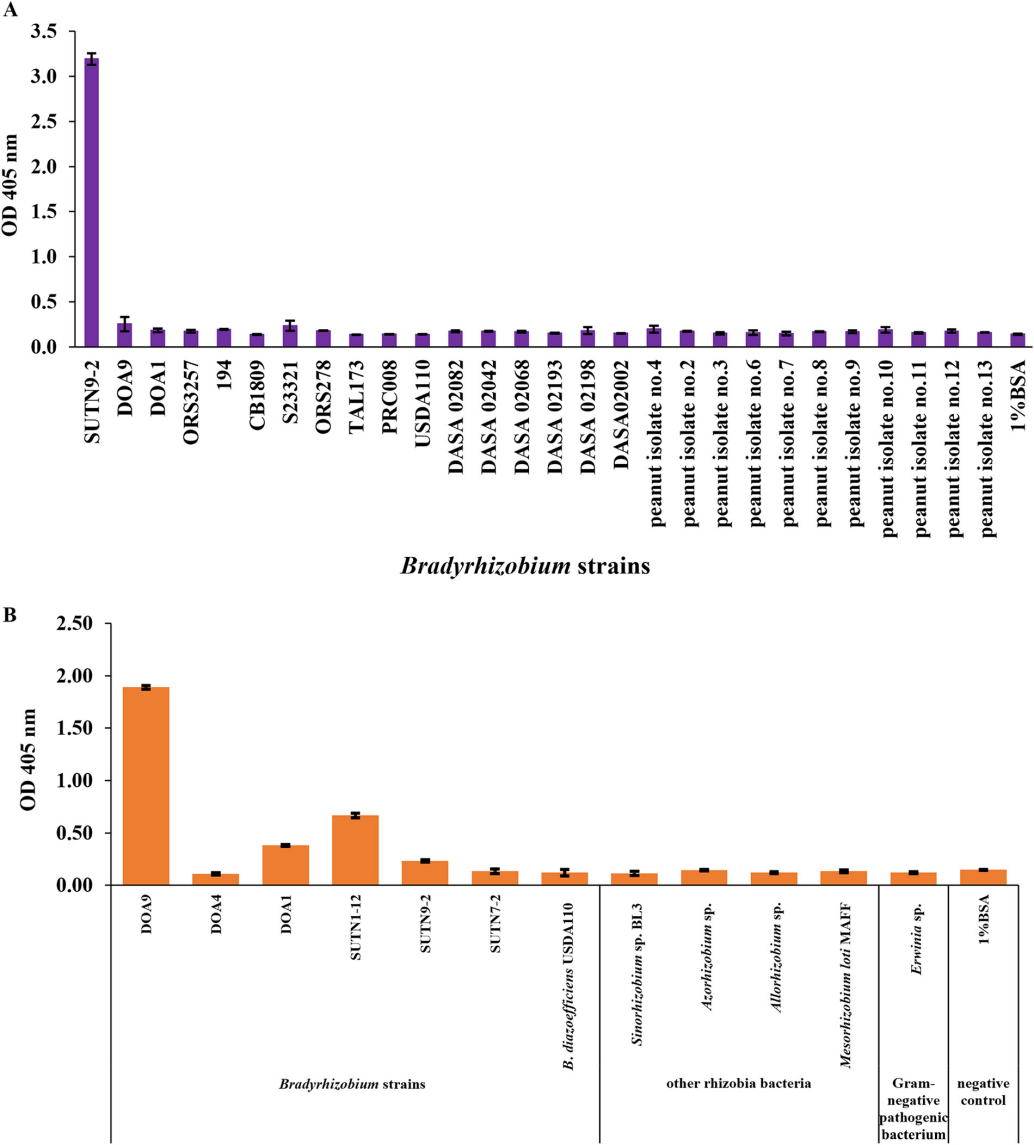
Cross reactivity of scFv antibody by ELISA. Binding activities of yiN92-1e10 (A) and yiDOA9-162 (B) scFv were determined by ELISA on a plate coated with 5 μg of various target antigens as indicated in the graphs. Boiled bacterial targets for yiN92-1e10 scFv (A) are *Bradyrhizobium* strains, SUTN9-2, PRC008, USDA110, DOA9, DOA1, ORS3257, 194, CB1809, S23321, ORS278, TAL173, DASA02002, DASA02082, DASA02042, DASA02068, DASA02193, DASA02198, peanut bradyrhizobia isolates no. 2, 3, 4, 6, 7, 8, 9, 10, 11, 12, 13 and 1% BSA. Boiled bacterial targets for yiDOA9-162 scFv (B) are *Bradyrhizobium* strains DOA9, DOA4, DOA1, SUTN1-12, SUTN9-2, SUTN7-2, B. diazoefficiens USDA110, other rhizobia bacteria (*Sinorhizobium* sp. BL3, *Azorhizobium* sp., *Allorhizobium* sp., Mesorhizobium loti MAFF), *Erwinia* sp. (pathogenic bacterium), and 1% BSA. The wells were detected with 5 μg of scFv antibody. One percent BSA was served as negative control. Values are the mean of triplicate wells. Error bars show the standard deviation for each set of data.

To evaluate the binding specificity of the two anti-bacterial scFv antibodies, the ELISA against boiled cell bacteria was performed. The binding of yiN92-1e10 scFv was investigated against 27 other related *Bradyrhizobium* strains and yiDOA9-162 scFv was tested against 11 other related strains by ELISA. The boiled bacterial cell (5 μg/well) were immobilized and incubated with 5 μg/well of the scFv clones. Specific binding of yiN92-1e10 to *Bradyrhizobium* strain SUTN9-2 and yiDOA9-162 to *Bradyrhizobium* strain DOA9, respectively, could be obtained as demonstrated in Fig. 4A and B. Anti-SUTN9-2 scFv (yiN92-1e10) did not cross-react with any of the related *Bradyrhizobium* strains tested. The absorbance signals from ELISA of the tested strains (PRC008, USDA110, DOA9, DOA1, ORS3257, 194, CB1809, S23321, ORS278, TAL173, DASA02002, DASA02082, DASA02042, DASA02068, DASA02193, DASA02198, and peanut isolates no. 2, 3, 4, 6, 7, 8, 9, 10, 11, 12, and 13) were not significantly different from the negative control, 1% BSA (signal values range between 0.1 and 0.3), while those against SUTN9-2 was as high as 3.2 (OD_405_ nm) (Fig. 4A). Anti-DOA9 scFv (yiDOA9-162) could not bind to 10 out of 11 other related strains but can cross-react with *Bradyrhizobium* strain SUTN1-12. However, the binding signal of yiDOA9-162 against SUTN1-12 (0.7 signal value) was 2.5 times lower than DOA9 (1.9 signal value) (Fig. 4B). These results indicated that strain specific scFv antibodies against *Bradyrhizobium* SUTN9-2 and DOA9 could be generated.

### Detection of SUTN9-2 in rice after inoculation.

**FIG 5:**
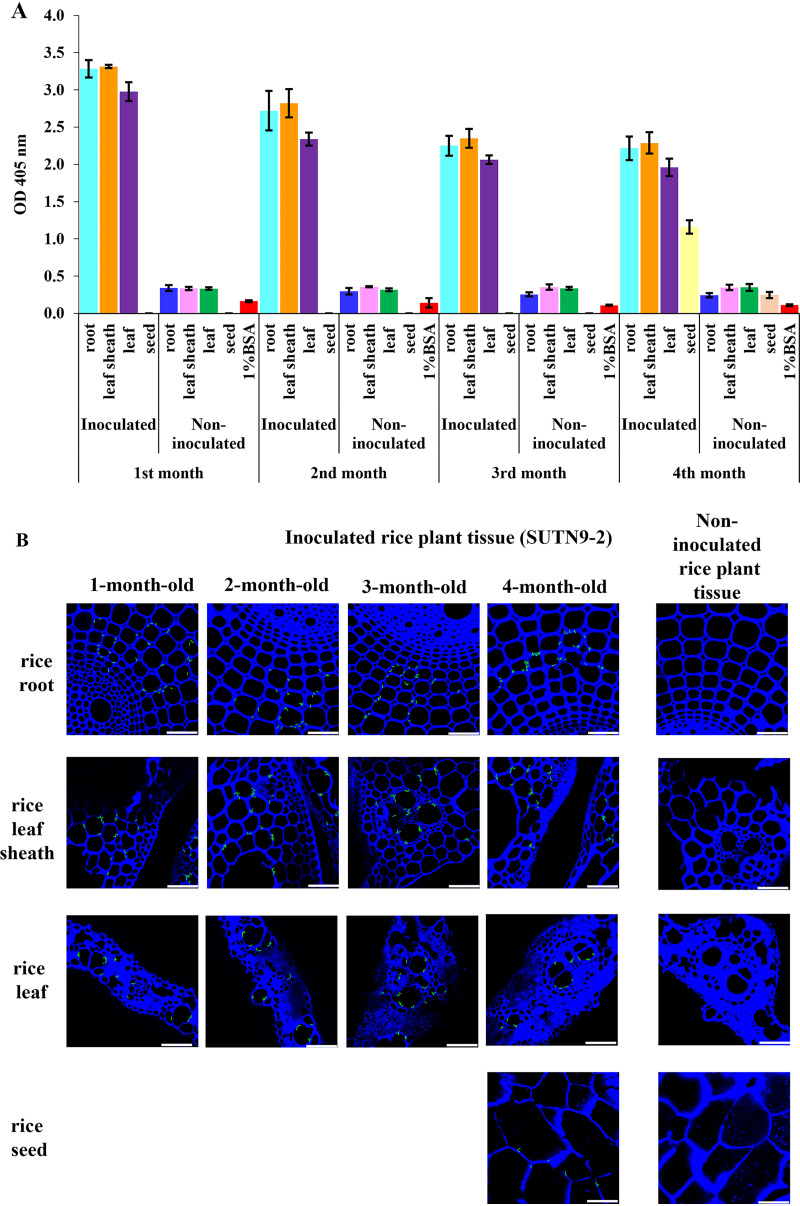
Endophytic colonization of rice root, leaf sheath, leaf and seeds by *Bradyrhizobium* strain SUTN9-2. Detection of bacterial cells from extracts of rice root, leaf, leaf sheath and seeds by ELISA (A). The values are the mean of triplicate wells. Error bars show standard deviation for each set of data. Confocal laser scanning micrographs of rice root, leaf, leaf sheath and seed inoculated with SUTN9-2 at 1st, 2nd, 3rd, and 4th month after cultivation (B). Cross-section of the tap root, flag leaf, leaf sheath above the stem base and seeds were examined as indicated in each figure. Scale bar is 50 μm at ×600 magnification.

To further investigate the possibility of applying the scFv antibody for monitoring the bacteria in a simulated ecosystem, the scFv antibody against *Bradyrhizobium* strain SUTN9-2 was selected for further study since the SUTN9-2 strain can form symbiotic nodules with many of the *V*. *radiata* cultivars (22) and effectively promote rice growth as endophyte (4). In this study, rice was grown in pots under greenhouse conditions, and the presence of *Bradyrhizobium* SUN9-2 as an endophyte in the inoculated rice seeds, leaves, leaf sheath and roots was investigated at different growth stages (1, 2, 3, and 4-months after inoculation) by ELISA and confocal immunofluorescence staining. As illustrated in Fig. 5A, *Bradyrhizobium* SUTN9-2 cells could be detected by ELISA with specific scFv yiN92-1e10 in the extract of rice leaves, leaf sheath, root, and seed at various time points (first, second, third, and fourth month after rice cultivation in the pots). The ELISA signals from each rice tissue were not significantly different at various time points. The bacteria can be found in roots, leaf sheaths, and leaves, with a slightly more abundance in leaf sheath than leaf. The endophytic SUTN9-2 were also detected in rice seeds after the rice was harvested (fourth month after inoculation). The lowest ELISA signal was observed in the seeds when compared with other rice plant tissues at harvest time (4 months).

In addition to ELISA, scFv yiN92-1e10 could also be used to observe the rod-shaped endophytic bacteria in the intercellular and intracellular spaces of root, leaf sheath, leaf, and seed of rice at different time points (Fig. 5B) using confocal laser scanning immunofluorescent microscopy. No antibody specific-bacterium was detected in the noninoculated rice plant tissues.

### Investigation of SUTN9-2 nodulation in mung bean using rice stubble as inoculum.

**FIG 6:**
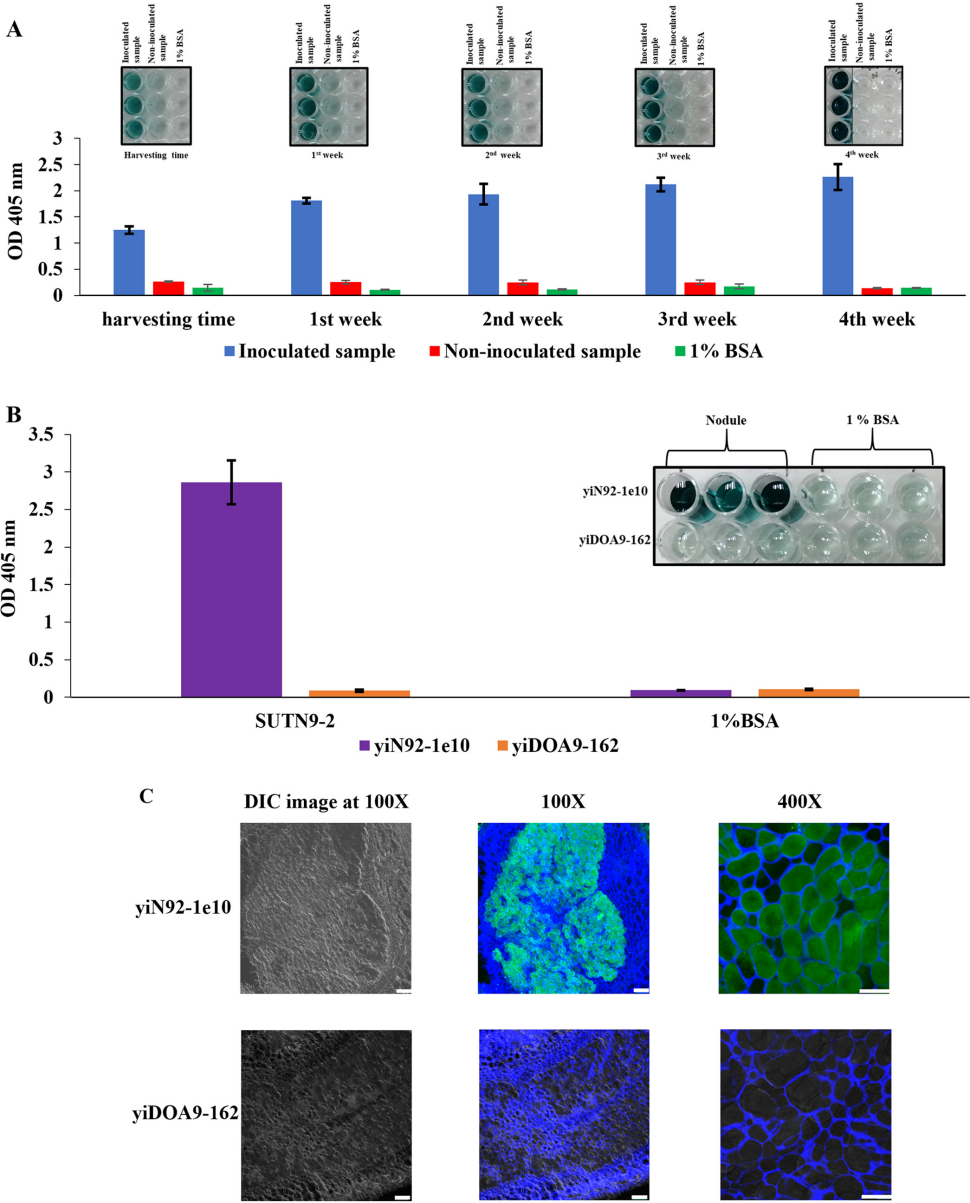
Persistence and nodulation of *Bradyrhizobium* SUTN9-2 from rice stubbles in mung bean. ELISA of bacterial cells from soil suspension (A) and bacteroid from nodule (B). The values are the mean of triplicate wells. Error bars show the standard deviation for each set of data. (C) Confocal laser scanning micrographs of nodule inoculated with SUTN9-2. The cross sections of nodule were stained with scFv yiN92-1e10 and yiDOA9-162. Green spot indicated the green-fluorescent staining of scFv antibody, using secondary antibody conjugated with anti-His Dylight 488. Plant cell walls were stained with a blue fluorophore (Calcofluor white M2R) and emitted blue color. The bacteroides are shown as green spots inside a blue plant cavity. The nodule images observed by differential interference contrast (DIC) technique were also indicated. Scale bar is 100 μm at ×100 magnification, and 50 μm at ×400 magnification.

To complete the observation of legume-rice-legume cropping system, scFv yiN92-1e10 was used to detect SUTN9-2 in soil samples at different time points by ELISA (Fig. 6A). From the time of rice harvest until 4 weeks later, the amount of SUTN9-2 in the soil gradually increased, corresponding with an increase in the ELISA signal. Only background value was detected in noninoculated soil. To simulate the rotational cropping system, after 4 weeks, mung bean seeds were planted into each pot. The nodulation was observed on the root of the mung bean at 28 days after cultivation. The symbiotic *Bradyrhizobium* strain SUTN9-2 inside the nodule could be detected with specific scFv yiN92-1e10 by ELISA (Fig. 6B) and confocal immunofluorescence staining (Fig. 6C). No signal was detected when nonspecific scFv yiDOA9-162 was used in both assays. These results revealed that SUTN9-2 persisted in rice tissues until rice-harvest season and could be used as inoculum for mung bean in the next season in the rotational cropping system and the scFv yiN92-1e10 will be a useful tool for monitoring the persistence of bradyrhizobial inoculum under this cropping system.

### Determination of nodule occupancy by scFv antibody immunofluorescence staining.

**FIG 7:**
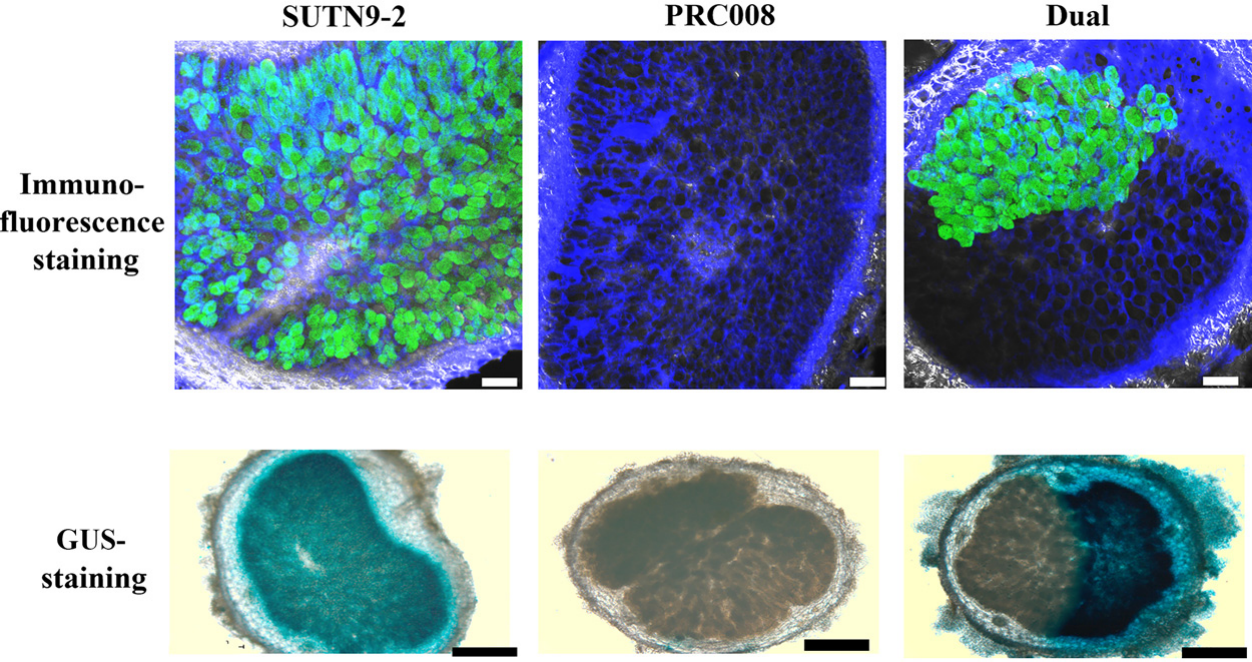
Bioimaging of nodule occupancy. Nodule phenotype after co-inoculation with SUTN9-2 (wild type) and PRC008 (wild type) for immunofluorescence staining (upper panel), and with SUTN9-2 (GUS-tagged strain) and PRC008 (wild type) for GUS-staining (lower panel). Green spots indicated the green-fluorescent staining of scFv yiN92-1e10 antibody, using secondary antibody conjugated with anti-His-Dylight 488. Blue color indicated GUS-staining of SUTN9-2 (SUTN9-2 GUS reporter gene-tagged strain) using the X-Gluc substrate. Scale bar is 100 μm and 500 μm at ×100 magnification for immunofluorescence staining and GUS-staining, respectively.

Finally, the possibility of using of recombinant scFv yiN92-1e10 antibody to study symbiotic bacteroid nodule occupancy was investigated in comparison with the standard GUS-staining assay. Mung beans were inoculated with *Bradyrhizobium* sp. SUTN9-2 wild type or GUS-tagged strain, and PRC008 wild type (single inoculation) or a combination of SUTN9-2 wildtype or GUS-tagged and PRC008 at 1:1 ratio (co-inoculation). After 30 days, the nodules were observed under confocal laser scanning microscope for immunostaining of wild type SUTN9-2 or a light microscope for GUS-tagged strains. Green spots indicated the green-fluorescent staining of scFv yiN92-1e10 antibody, using secondary antibody conjugated to anti-His-Dylight 488. Using this immunofluorescence staining, the nodule area occupied by SUTN9-2 (wild type) exhibiting the green-fluorescent color was easily distinguished from the area occupied by PRC008 (without fluorescence) in the dual-occupied nodules (Fig. 7, right panel). For GUS-staining assay, the area occupied by SUTN9-2 GUS-tagged strain appeared blue from the β-galactosidase substrate X-Gluc. Quantification of nodule occupancy (Table 2) indicated that 59.7 or 60.7% of the nodules were co-occupied by both *Bradyrhizobium* strains, when determined using scFv staining or GUS assay, respectively. Moreover, the results also suggested that the PRC008 strain was more competitive than SUTN9-2 in nodule formation. The percentage of single nodule occupied by SUTN9-2 and PRC008 was 9.2 and 31.1%, respectively, when determined by immunostaining. For GUS-staining, the percentage of one single nodule occupied by SUTN9-2 and PRC008 was 13.2 and 26.1%, respectively. According to these results, it can be concluded that recombinant scFv antibody is a valuable reagent for the study of symbiotic nodule formation in legumes.

**TABLE 2:** Immuno- and GUS-staining for the analysis of mung bean nodule occupancy by SUTN9-2 and PRC008^*a*^

Plant inoculated with	Nodule occupancy %					
	Immunostaining			GUS-staining		
	SUTN9-2	PRC008	Co-occupied	SUTN9-2	PRC008	Co-occupied
SUTN9-2	100 ± 0	0 ± 0	0 ± 0	100 ± 0	0 ± 0	0 ± 0
PRC008	0 ± 0	100 ± 0	0 ± 0	0 ± 0	100 ± 0	0 ± 0
SUTN9-2 and PRC008 (1:1)	9.2 ± 3.0	31.1 ± 11.6	59.7 ± 9.9	13.2 ± 7.9	26.1 ± 15.5	60.7 ± 19.4

a All values are the mean of five replications.

## DISCUSSION

Phage display is a powerful technology for the generation of human monoclonal antibodies (13). The scFv format has several advantages over other antibody formats, namely, more efficient and stable for display on phage coats (23), higher permeability due to nano size (24), easy to express in E. coli (25) and other expression hosts (26). It can also be further engineered to suit various assay formats, including biosensor-based detection (15). Another key advantage of phage display antibody technology is the possibility for high-throughput production of recombinant antibody against a wide variety of targets of interests at the same time (27).

Our research demonstrates, for the first time, the benefits of using a compact human phage display scFv antibody library for rhizobium research. The antibody library in this study was created from a healthy population that had previously encountered a myriad of antigens in the region of northeastern Thailand around 15 years ago, apparently from this study, including nitrogen-fixing soil bacteria. Specific scFv antibodies from this library has been generated against a wide variety of targets such as mycotoxins (15, 25), venom (28), pathogenic bacteria (17), as well as virus (29) and cancer biomarker (30). From our previous study, the recombinant scFv antibody that could bind specifically to *Bradyrhizobium* strain DOA9 was successfully generated (18), from a phage-displayed rabbit scFv antibody library, created from an immunized rabbit. In this study, specific scFv antibodies against two *Bradyrhizobium* strains, DOA9 and SUTN9-2, could be obtained from a naive human phage library without utilization of experimental animals. The isolated scFv antibody can bind to the planktonic bacteria (both boiled and nonboiled), the bacteroid form inside plant nodule, and endophyte inside the rice tissue. Since the antibody in the scFv format is small and monovalent, the concentration of the antibody that was used to detect the antigen was relatively high (1- 5 μg/ml), when compared to commercial monoclonal or polyclonal antibodies. However, the antibody in an scFv format can be produced easily from an E. coli expression system. Moreover, it is possible to convert scFv antibody to Immunoglobulin G (IgG) or other formats for various detection methods in the future (31). The optimal concentration of the scFv antibody used in ELISA experiments can varied greatly, depending on different antibody clone, which can be determined by checkerboard titration experiment (17).

So far, there have been a few reports on the identification and characterization of traditional monoclonal antibodies against Rhizobia surface antigens. Eight monoclonal antibodies against Rhizobium leguminosarum 3841 could bind with either core oligosaccharides component or lipid A moiety of lipopolysaccharide (LPS) with different degree of cross-reactivity with another strains (32). Several monoclonal antibodies were generated against Rhizobium meliloti and its mutants for discriminative typing (33). Production and epitope analysis of several monoclonal antibodies against LPS of Rhizobium leguminosarum biovar *trifolii* strains have been reported and their epitope have been determined (34). However, none of these demonstrated the applications of using monoclonal antibodies to detect the bacteria after physiological changes in nodules or monitor rhizobia in field applications. *Bradyrhizobium* sp. usually contain extracellular polysaccharide (EPS), capsular polysaccharides (CPS) or K-antigen, flagella proteins or H-antigen, and LPS or O-antigen (35). Since K and H-antigens are heat labile (36), the candidate epitope of this selected antibodies is likely to be present on an O antigen or another strain specific cell surface molecule. In our study, the antigen on the bacterial cell surface, which seems to be heat stable and dominant in all forms of this diazotroph will be identified in the next step. This information will be valuable in understanding the mechanism of specificity in legume-rhizobium interactions and the evolution of rhizobium symbiosis.

Although, both *Bradyrhizobium* strains SUTN9-2 and DOA9 are rice endophytes, DOA9 cannot promote rice growth effectively (37). Therefore, strain SUTN9-2 was chosen as inoculated strain in rice-legume rotational cultivation. Determination of cross-reactivity to related *Bradyrhizobium* strains (both type strains and indigenous strains) revealed that scFv yiN92-1e10 has high specificity with *Bradyrhizobium* sp. SUTN9-2. The high specificity of this recombinant yiN92-1e10 antibody indicated that it could be used to precisely distinguish *Bradyrhizobium* strain SUTN9-2 from other related strains in the environment. Therefore, it should be appropriate for routine quality control and monitoring persistence of SUTN9-2 inoculum as biofertilizer for sustainable and precision agriculture of rice-legume rotational cropping system. Using the scFv yiN92-1e10 antibody, the strain SUTN9-2 could be observed as endophyte in both the intercellular and intracellular spaces of rice tissues at different growth stages. This result is in accordance with previous report on the presence of endophytic R. leguminosarum bv. *trifolii*, recovered from surface-sterilized leaf sheaths, leaves, and roots (38). Our results also confirmed previous observation that endophytic rhizobia persisted in rice throughout the rice growing season and could infect the rotational crop, mung bean (4).

While the strain DOA9 was not the focus of this study, because it could not promote the grown of mung bean and rice, this strain could effectively symbiose with Aeschynomene americana, of which its symbiosis interactions have not been much elucidated. Therefore, binding characteristic of antibody yiDOA9-162 will be further investigated in the future work dealing with the symbiosis in A. americana.

Previous studies on the evaluation of nodule occupancy was done using reporter genes such as *gfp* and *gusA* (39). Although this method is effective, it requires generation of genetically modified organisms (GMOs) expressing *gfp* and/or *gusA* genes at regions that would not disrupt bacterial growth and nodulation capacity. Moreover, GMOs have always been considered a threat to the environment and human health (40) and need to be regulated under the general statutory authority of environmental, health, and safety laws. Our study demonstrated that the efficacy of immunofluorescence staining and GUS-staining methods was not significantly different for the determination of nodule occupancy percentage (Table 2). Accordingly, using specific recombinant scFv antibody that bind specifically against relevant *Bradyrhizobium* strains is a more convenient method that can replace the use of GUS reporter system. Furthermore, the functionality of the recombinant antibody can be further improved to suit various applications, including an increase in affinity, and stability (41). These improvements will also allow easy and rapid quantification of the population of rhizobia bacteria in an inoculum or ecosystem, especially where the GMOs are not allowed to apply in the fields. The detection format could be in the form of rapid and simple test kit or biosensor-based, for commercialization as point-of-demand diagnosis in the agricultural fields (42).

In conclusion, a convenient method based on phage display antibody technology was successfully employed for the generation of specific recombinant antibodies: yiN92-1e10 and yiDOA9-162 for the detection of *Bradyrhizobium* strains SUTN9-2 and DOA9, respectively. Application of recombinant yiN92-1e10 scFv for monitoring *Bradyrhizobium*-legume symbiosis in simulated legume-rice rotational cropping system was also demonstrated. This approach is a powerful tool to allow scientists around the world to study symbiosis in nature and actual rotational cropping situation in the fields. This finding is essential because current method to study symbiosis must be done with GUS-tagged bacteria, which is a GMO, and is not allowed in many regions of the world. Monitoring of the bacteria using antibody generated by our approach is, therefore, critical for understanding of real situation outside experimental setting in a greenhouse, when competitions from other soil bacteria in the field can't be simulated. Raising monoclonal or polyclonal using traditional methods would not be possible because there are so many diazotrophs on the planet. Phage display is a powerful tool for high-throughput generation of recombinant antibodies and the technology are advancing (27). Obtaining recombinant antibody from phage display against myriad of soil bacteria will allow monitoring of diverse bacteria in different ecosystem. Therefore, this methodology can be further employed for the study of other plant-microbe interactions and monitoring of biofertilizer in diverse sustainable cropping systems as well as in precision agriculture. The production of highly specific recombinant antibodies might proof valuable for applied agronomical microbiology, even beyond plant growth-promoting bacteria and (Brady)rhizobium-legume symbioses.

## MATERIALS AND METHODS

### Materials.

All chemical reagents were molecular biology grade. Phage display scFv library from nonimmunized human, Yamo I was constructed in our laboratory using B-lymphocytes from 140 healthy individuals in the Northeastern Thailand (12). E. coli TG1 was obtained from MRC, Cambridge, and was used for cloning and amplification of phages. E. coli SHuffle T7 Express (New England Biolabs [NEB], USA) was used for protein expression. The anti-M13/horseradish peroxidase (HRP) and His-probe-HRP were purchased from Amersham-Pharmacia Biotech (Uppsala). Anti-His-Dylight 488 secondary antibody and Calcofluor white stain were purchased from Abcam and Sigma, respectively. *Bradyrhizobium* strains SUTN9-2, DOA9 and other *Bradyrhizobium* strains were obtained from School of Biotechnology, Suranaree University of Technology.

### Preparation of pure culture antigen.

*Bradyrhizobium* strains SUTN9-2 and DOA9 were subcultured on Yeast extract Mannitol (YM) Agar medium. After 5 days, the single colony was picked into 20 ml YM broth. Thereafter, the bacteria were grown at 28°C, 180 rpm for 7 days. The cells were collected by centrifugation at 3,300 *g*, 4°C for 15 min, and resuspended in sterile saline buffer (0.85% wt/vol of NaCl). After that, the same number of cells were adjusted to 10^9^ cells/ml by measuring the optical density of the suspension on the spectrophotometer at an optical density of 1 (OD_600_ nm). Then, the cell suspension was split up and centrifuged at 3,300 *g*, 4°C for 15 min and treated under boiled, and nonboiled (whole cell) conditions. For boiled cell treatment, the cells were suspended in 0.85% (wt/vol) NaCl of the same volume and boiled in water bath at 100°C for 1 h. For nonboiled condition, the cells were suspended in 100 mM NaHCO_3_ of the same volume used for washing. Then, the total protein was measured by bicinchoninic acid assay (43). The stock of pure antigen was stored at −20°C.

### Preparation of nodule antigen.

The seeds of mung bean (Vigna radiata) and shyleaf (Aeschynomene americana) were surface sterilized and germinated. The germinated seeds were then transplanted into plastic pouches containing N-free nutrient solution (36). The bacterial suspension of SUTN9-2 and DOA9 (10^9^ colony forming unit, CFU/ml) was inoculated onto each seedling of V. radiata and A. americana, respectively, at 2 days after transplanting. Plants were grown under controlled environmental condition of 28 ± 2°C, 70% relative humidity on 16/8 h day/night cycle for 1 month. The root nodules and bacteroid suspension were used for further analyses as antigen by immunostaining and ELISA techniques.

### Biopanning against *Bradyrhizobium* strains SUTN9-2 and DOA9.

Selection was performed using *Bradyrhizobium* strains SUTN9-2 and DOA9 as targets. Two Maxisorp immuno tubes (Nunc, Denmark) were coated separately with 20 μg of boiled *Bradyrhizobium* strains, SUTN9-2 and DOA9, in sodium carbonate buffer at 4°C overnight. After that, the immuno tubes were washed three times with phosphate-buffered saline (PBS) and blocked with PBS containing 2% (wt/vol) skim milk (2% MPBS) for 2 h at room temperature. The tubes were washed three times with phosphate-buffered saline (PBS) before adding 10 μl (10^11^ PFU/μl) of phage library in 290 μl of 2% (wt/vol) MPBS and then incubated at room temperature for 2 h. Unbound phage was washed away with five times PBS supplemented with 0.1% (vol/vol) Tween 20 (PBST) and followed by 5 times PBS. Phage antibodies against each *Bradyrhizobium* strain was eluted with 1 μg/ml of trypsin buffer, followed by 0.1 M glycine-HCl, pH 2.0. To obtain individual phage clones, the eluted phage was infected with E. coli TG1 and incubated at 37°C for 30 min without shaking. Then, infected cells were subjected to 10-fold-serial dilution (10^1^-10^4^) and spread on 2x YT agar plates supplemented with 100 μg/ml ampicillin and 1% (wt/vol) glucose. The agar plates were incubated overnight at 37°C. Each single bacterial colony represents individual phage clone. One round of selection was carried out for both strains.

### Individual Phage Rescue.

Individual phage-infected colonies were randomly picked from the 2x YT plate and grown in wells of a 96-well plate (Nunc, Denmark) containing 100 μl 2× YT, 100 μg/ml ampicillin and 1% (wt/vol) glucose. After overnight incubation at 37°C, small inocula (10 μl) from each well were transferred to a second 96-well plate containing 400 μl of 2x YT plus 100 μg/ml ampicillin and 1% (wt/vol) glucose. The first plate was kept as a master stock by adding glycerol to a final concentration of 20% (vol/vol) and kept at −20°C. The second plate was incubated with shaking at 37°C for 3 h, and 10^10^ PFU/ml of KM13 helper phage was added to each well to rescue the recombinant phages. The plates were then incubated at 37°C for 1 h without shaking and centrifugation at 3,300 *g* for 10 min. The supernatant was discarded, and the pellet was resuspended in 400 μl of 2x YT containing 100 μg/ml ampicillin and 50 μg/ml kanamycin and cultured at 30°C for 20 h with shaking (250 rpm). The overnight culture was spun at 3,300 *g* for 10 min, and 100 μl of the supernatant-containing phage was used in monoclonal phage ELISA.

### Phage ELISA against pure bacterial culture.

ELISA was performed according to the protocol described by Vu et al. (18). The Immuno 96 microWell plate (Nunc, Denmark) was coated with 5 μg of boiled and nonboiled pure culture antigen of *Bradyrhizobium* strains SUTN9-2 and DOA9 at 4°C overnight. After that, the plate was washed 3 times with PBS and blocked with PBS containing 2% (wt/vol) skim milk (MPBS) for 1 h. One hundred microliters of phage supernatant and 50 μl of 4% (wt/vol) MPBS were added to each well and incubated at room temperature for 2 h. After incubation for 2 h, the plate was washed 3 times with PBST and 2 times with PBS. Subsequently, 100 μl of horseradish peroxidase-labeled anti-M13 1:5,000 dilution in 2% (wt/vol) MPBS) was added into each well. After incubation at room temperature for 1 h, the wells were washed again, as described previously, and 200 μl of substrate solutions ABTS (2,2′-azinobis [3-ethylbenzthiazolinesulfonic acid, Sigma]) were added into each well and incubated at room temperature for 30 min. The reaction was quantified by measuring the absorbance at 405 nm in an ELISA plate reader (Sunrise, TECAN, Austria).

### DNA sequence analysis and 3D structure prediction.

Plasmids from positive phage clones were extracted using a DNA miniprep kit (Qiagen, Germany) and the DNA sequences were determined by automated DNA sequencing (Macrogen, Korea), using primers pMOD5’: 5′ CAG GAA ACA GCT ATG ACC 3′, and pMOD3’: 5′ CCC TCA TAG TTA GCG TAA CG 3′. The DNA sequence was analyzed with IgBLAST and the complementarity determining regions (CDRs) 1, 2, 3 were identified with IMGT software. For 3D structure modeling, the nucleotide sequence was translated to an amino acid sequence using ExPASy website. Homology modeling of the three-dimensional (3D) structures of yiN92-1e10 and yiDOA9-162 scFv antibodies was generated from the amino acid sequences using the SWISS-MODEL website. The server chose the template by sequence identity analysis. Thereafter, the sequence was processed by the server for modeling. The templates with the 79.57 and 70.76% sequence identity were chosen among three generated models from yiN92-1e10 and yiDOA9-162 scFv antibodies, respectively. Models were visualized with the program PyMOL (www.pymol.org).

### Cloning and expression of scFv antibodies.

For larger scale production of soluble scFv antibodies, the gene encoding for scFv antibody clones: yiN92-1e10 and yiDOA9-162 were digested with NcoI and NotI restriction enzymes and sub-cloned into pET-21d (+) vector (NEB, USA), to generate pET21d+/yiN92-1e10 and pET21d+/yiDOA9-162 plasmids. The DNA of scFv fragments and pET-21d (+) vector were digested with the NcoI (20U/μl, NEB, USA) and NotI (20U/μl, NEB, USA) enzymes. The digestion reactions of scFv fragments and pET-21d (+) vector were performed separately, each in a total volume of 50 and 100 μl, respectively. The reaction mixtures consist of 10 μg of insert DNA, 2 μg of vector DNA, 1× Cutsmart buffer, 20U of NcoI (20U/μl; NEB, USA) and 20U of NotI (20U/μl; NEB, USA). The reactions were incubated at 37°C for 16 h and heat inactivated at 80°C for 20 min. Afterwards, the digested DNA of vector was dephosphorylated by adding 1 μl of CIP enzyme (10U/μl; NEB, USA) and incubated at 37°C for 1 h. The inserts and vector were separated from stuffer fragments by gel electrophoresis and followed by Wizard clean up kit (Promega, USA). The DNA of scFv antibodies were ligated into pET-21d (+) vector at a 3:1 ratio and incubated at 16°C for 16 h. The ligation reaction was then transformed into E. coli DH5α by chemical transformation. Subsequently, the transformed cells were spread on LB plate containing 100 μg/ml of ampicillin and incubated overnight at 37°C. The single colony was picked and analyzed by double digestion with NcoI and NotI restriction enzymes. Plasmids were purified from each clone (Qiagen spin Miniprepkit, USA). The integrity of the constructs was confirmed by automated DNA sequencing (Macrogen, Korea), using universal primer (T7 promoter and T7 terminator).

To express yiN92-1e10 and yiDOA9-162 soluble scFv, pET21d+/yiN92-1e10 and pET21d+/yiDOA9-162 plasmids were transformed into E. coli SHuffle T7 Express. After that, a single colony containing each plasmid was inoculated into 5 ml of LB (Luria-Bertani) media containing 100 μg/ml of ampicillin and cultured overnight at 30°C. Four milliliters of overnight culture were inoculated into 200 ml of LB medium containing 100 μg/ml of ampicillin. Cells were cultured at 30°C until the OD_600_ nm reach 1.0, before induction with 1.0 mM IPTG (isopropyl-β-d-thiogalactopyranoside) and further incubated at 16°C for 24 h. This expression condition was an optimized condition for the expression of yiN92-1e10 and yiDOA9-162 scFv antibodies, according to the standard guideline of E. coli SHuffle T7 Express strain datasheet (NEB, USA).

To harvest and purify the recombinant antibodies, the cell pellets were harvested by centrifugation at 10,000 *g* for 10 min, then re-suspended in 20 ml of ice-cold lysis buffer (20 mM sodium phosphate, 500 mM NaCl, and 45 mM imidazole, pH 7.4) containing 1 mg/ml of lysozyme. Cells were disrupted by intermittent sonication for 10 min on ice using 30 s pulse and 30 s break for cooling, followed by centrifugation at 4°C for 30 min at 10,000 *g*. The retained soluble fractions were further processed for protein purification. The supernatant was applied to 1 ml His-Trap column (GE Healthcare, USA) pre-equilibrated with the binding buffer (20 mM sodium phosphate, 500 mM NaCl and 45 mM imidazole, pH 7.4). The soluble scFv was eluted with 250 mM imidazole in an elution buffer (pH 7.4). Fractions containing yiN92-1e10 and yiDOA9-162 scFv were subjected to buffer exchange by dialysis with PBS buffer at 4°C. The samples were collected and kept at 4°C. The eluted fractions and purity of the samples were assessed by SDS-PAGE. The binding affinity of soluble scFv antibodies was determined by scFv ELISA and immunostaining assay with both types of antigen, that is, pure culture (boiled and nonboiled) and nodule samples.

### Detection of SUTN9-2 and DOA9 with scFv antibodies by ELISA.

ELISA was performed according to previously published protocol (18). Both boiled and nonboiled bacterial antigens of SUTN 9-2 and DOA9 were diluted in sodium bicarbonate buffer to achieve 5 μg of total protein per well. One percent (wt/vol) BSA in PBS was used as blank. The bacteria samples were immobilized overnight on the 96-well ELISA plate (MicroWell Nunc) at 4°C. Then, the wells were washed two times with PBS and blocked with 2% (wt/vol) MPBS for 1 h at room temperature. After blocking, the wells were rinsed two times with PBS. Then, 5 and 10 μg of two scFv antibodies were added into each well. The scFv antibody 3E3, against aflatoxin B_1_ (21) was used as a negative control. The binding was done at room temperature for 1 h. Then, the wells were washed three times with PBST (PBS with 0.05% Tween 20) followed by two times washing with PBS. Bound scFv antibodies were detected with His-probe HRP (horseradish peroxidase) (1:5,000 dilution PBS). After incubating at room temperature for 1 h, the wells were washed again three times with PBST and two times with PBS. Color reaction was developed by adding 200 μl ABTS (2,2′-azinobis [3-ethylbenzthiazolinesulfonic acid]) peroxidase substrate containing 0.05% H_2_O_2_. Detection was done by measuring the absorbance at 405 nm in an ELISA plate reader (Sunrise, TECAN, Austria). The assay was performed in triplicates.

To prepare plant nodules for ELISA, five nodules was detached from *V*. *radiata* and A. americana root inoculated with SUTN9-2 and DOA9, respectively. The nodule samples were washed with sterile water and then 5 nodules were gently ground in 1 ml of sodium carbonate buffer using sterilized small mortar and pestle. Then, 200 μl of the bacteroid suspension was added to each well of an ELISA plate. The assay was performed in triplicate as described for pure culture samples.

### Determination of scFv cross-reactivity.

To confirm binding specificity of recombinant scFv yiN92-1e10, ELISA was performed against *Bradyrhizobium* strain SUTN9-2 and other related 27 *Bradyrhizobium* strains, namely; PRC008, USDA110, DOA9, DOA1, ORS3257, 194, CB1809, S23321, ORS278, TAL173, DASA02002, DASA02082, DASA02042, DASA02068, DASA02193, DASA02198, and peanut bradyrhizobia isolates no. 2, 3, 4, 6, 7, 8, 9, 10, 11, 12, and 13. For recombinant scFv yiDOA9-162, ELISA was conducted against *Bradyrhizobium* strain DOA9 and other related 11 strains, namely; DOA4, DOA1, USDA110, SUTN1-12, SUTN9-2, SUTN7-2, *Sinorhizobium* BL3, *Azorhizobium* sp., *Allorhizobium* sp., Mesorhizobium loti MAFF, and *Erwinia* sp. One percent BSA was used as the negative control. The ELISA was conducted using boiled pure bacterial culture antigen as described above.

### Confocal immunofluorescent imaging of *Bradyrhizobium* SUTN9-2 and DOA9 with scFv antibodies.

The immunofluorescence analysis was performed according to previously published protocol (17). For pure culture, about 1 ml of broth culture was centrifuged (3,000 *g*, 5 min), washed once with 0.85% (wt/vol) NaCl, followed by a thorough rinsing with PBS, and then resuspended in PBS. About 5 μl of the suspension was spread into a smear on a glass slide and dried completely at 37°C. Subsequently, the smear was fixed with 4% paraformaldehyde and then blocked with 1% BSA-300 mM glycine-0.1% PBST for 30 min. After blocking, the cells were rinsed 3 times with PBS. Subsequently, 10 μg of scFv antibodies yiN92-1e10 and yiDOA9-162 was added per slide, respectively, at room temperature for 1 h. The slides were rinsed 3 times with PBS and incubated with 5 μl of 1:500 dilution of anti-His-Dylight 488 secondary antibody in the petri dishes for 1 h at room temperature. Unbound antibodies were removed by washing with PBS for 3 times. After that, the cells were incubated with 300 μM DAPI (4′,6-diamidino-2-phenylindole) for 5 min at room temperature and rinsed with PBS for 3 times. Finally, the cells were mounted with SlowFade gold antifade mountant (Invitrogen#S36936, USA) and then the slides were sealed. The images were acquired from the confocal microscope (Nikon A1, Japan).

For nodule samples, the cross sections at 65 μm thickness were cut using the vibratome and placed on the slides. Then, sections were incubated in PBS containing Calcofluor white M2R (Sigma) to a final concentration of 0.01% (wt/vol) for staining of the plant cell wall. After rinsing with PBS, the procedure was done as described above for pure culture samples.

### Application of scFv antibody for monitoring bradyrhizobial inoculum under rice-legume rotational cropping model.

The experiment was performed under pot trial condition. The plastic pots (10 L size, 25.5 cm diameter, and 22.5 cm height) were filled with 5 kg of low-organic-matter soil. Rice seeds were dehulled and surface disinfected by washing with 95% ethyl alcohol for 30 s, 10% hydrogen peroxide for 10 min, 3% sodium hypochlorite for 3 min, and then five to six times with sterilized water. Seeds were germinated in the dark at 30°C for 2 days on plates containing 0.8% (wt/vol) water agar. The germinated seeds were soaked overnight with a culture of *Bradyrhizobium* sp. SUTN9-2 (10^8^ CFU/ml). Then, the seedlings were transplanted into the plastic pots and grown in the greenhouse until harvest (seed maturation stage). The plants without inoculation served as control. Tap water was irrigated into each pot at 3 days interval. The scFv antibody was further applied for detection and monitoring in the following schemes.

### (i) Detection of rice endophytic bradyrhizobia by ELISA and immunostaining.

To monitor the endophytic bradyrhizobia in rice tissues, the 1, 2, 3, and 4-month-old rice tissues were washed with tap water and then surface sterilized with 70% ethanol for 1 min, 3% sodium hypochlorite for 3 min, then washed three times with sterilized water. Subsequently, the rice seeds, leaves, leaf sheath, and root were excised into small pieces and macerated separately with a sterilized mortar and pestle before dilution (1:1) in sodium carbonate buffer. After that, the rice extracts were passed through three layers comprising a miracloth (22-25 μm), membrane filter no.4 (25 μm) and no.1(11 μm) to remove the plant debris. Then, the endophytic bradyrhizobial cell in rice extracts were detected by ELISA as mentioned above.

For immunofluorescence assay, the cross sections of 85 μm thickness rice tissues were cut using the vibratome and placed on the slides. Then, sections were incubated in PBS containing Calcofluor white M2R (Sigma) to a final concentration of 0.01% (wt/vol) for staining of the plant cell wall. After rinsing with PBS, the immunostaining procedure was conducted as described above.

### (ii) Detection for the persistence of rice endophytic bradyrhizobia in soil samples.

After harvesting, the remaining rice stubbles were immediately incorporated into the soil in each pot. Then, the soil sample at 0, 1, 2, 3, and 4 weeks after harvest were mixed with sodium carbonate buffer at 1:1 ratio (wt/vol). All visible roots and debris were removed from the suspension. Then, the bradyrhizobia were detected by ELISA as described above. Soil sample in the pot containing noninoculated rice was used as a negative control.

### (iii) Detection of rice endophytic bradyrhizobia from nodules of mung bean.

After incorporating the rice stubbles into the soil for 4 weeks, mung bean seed was grown in each pot for 3 weeks under greenhouse conditions. The nodules from mung bean were collected and detected with specific scFv antibody by ELISA and immunofluorescence staining as described in the previous experiments.

### Quantification of nodule occupancy by scFv immunofluorescence staining.

The percentage of nodule occupancy was evaluated by immunofluorescence and GUS-staining techniques. For immunofluorescence staining, wild type of *Bradyrhizobium* strains SUTN9-2 and PRC008 were co-inoculated at the ratio of 1:1 into each seedling of mung bean. For GUS-staining, SUTN9-2 (GUS reporter gene-tagged strain) and wild type of strain PRC008 were co-inoculated as in the immunofluorescence staining set. Thereafter, plants were grown under controlled environmental condition of 28 ± 2°C, 70% relative humidity on 16/8 h day/night cycle (full light, 639 microeinsteins [μE]/m^2^/S) for 1 month. There were five replicates for each treatment in both staining methods. After 1 month, total nodule number from each plant was counted. The cross section of 65 μm thickness was cut from each nodule and stained. The immunofluorescence staining was performed as mentioned above. For the detection of GUS-marked rhizobia in plant nodule, the nodule sections were immersed in a microtiter plate containing the GUS assay solution (40 μl X-Gluc 20 mg/ml in N, N-Dimethyl-formamide, SDS 20 mg, methanol 2 ml, 1 M sodium phosphate buffer 0.2 ml and distilled water 7.76 ml), in vacuum for 120 min before overnight incubation at 28°C (44). Thereafter, the nodule was observed under light microscope and the occupancy percentage was calculated.
