# Supplementary material for: Application of Recombinant Human scFv Antibody as a Powerful Tool to Monitor Nitrogen Fixing Biofertilizer in Rice and Legume
Source: Microbiol Spectr. 2021 Dec 15;9(3):e02094-21. doi: 10.1128/Spectrum.02094-21 (PMC8672896; doi:10.1128/Spectrum.02094-21)
Supplement: SUPPLEMENTAL FILE 1 — Supplemental material. Download SPECTRUM02094-21_Supp_1_seq10.pdf, PDF file, 0.7 MB [file spectrum02094-21_supp_1_seq10.pdf]

Supplementary Materials for

Application of recombinant human scFv antibody as a powerful tool to monitor nitrogen fixing biofertilizer in rice and legume

Kyaut Kay Khaing, Kuntalee Rangnoi, Hanna Michlits, Neung Boonkerd, Nantakorn Teaumroong, Panlada Tittabutr, Montarop Yamabhai

Address correspondence to M. Yamabhai, montarop@g.sut.ac.th; P. Tittabutr, panlada@sut.ac.th

This PDF file includes:

Figs. S1 to S4

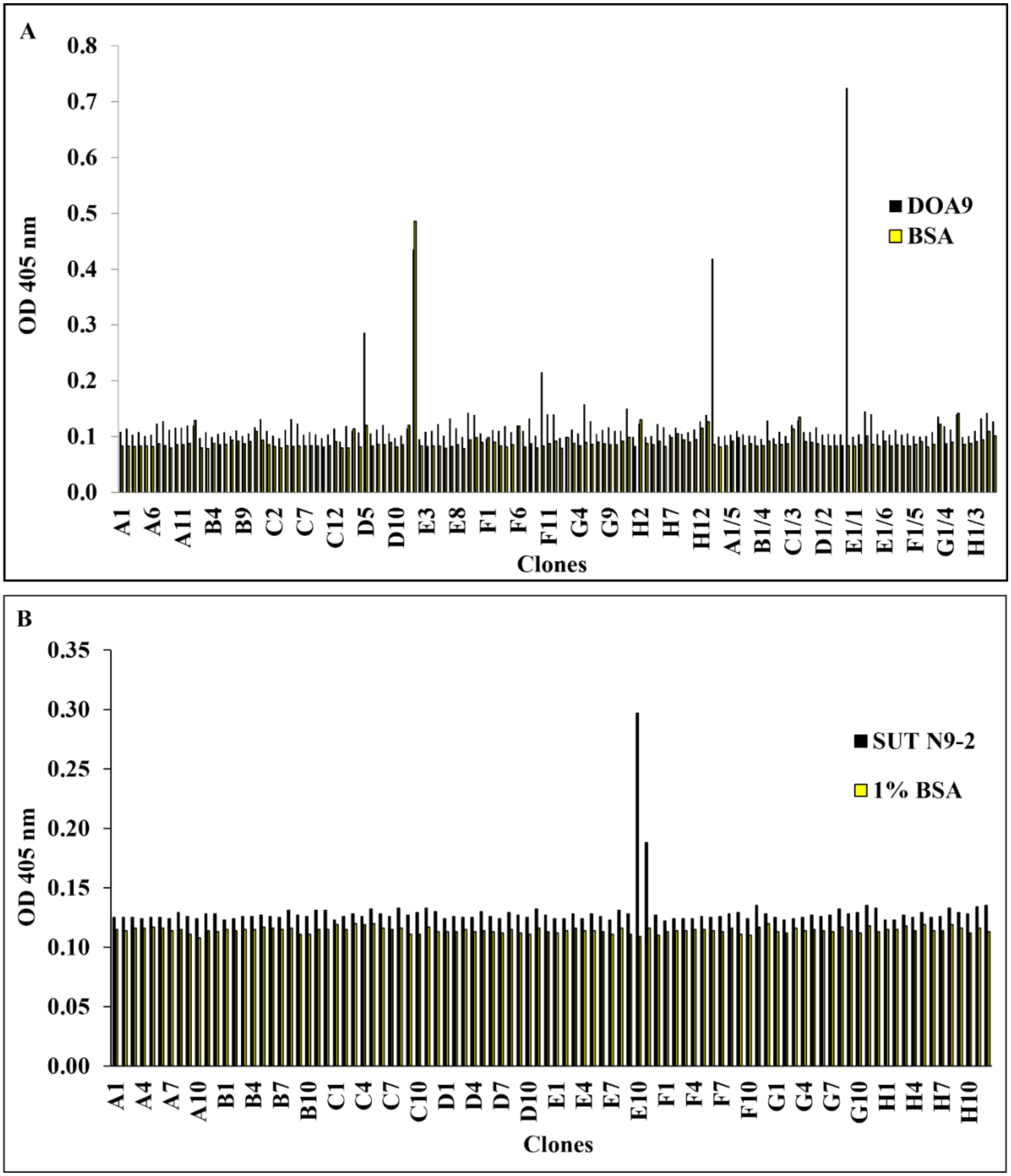

Figure S1. Confirmation of the binding of phage clones from the 1st round of panning. Phage ELISA was performed to confirm the binding of 144 and 96 selected phage clones against *Bradyrhizobium* strain DOA9 (**A**) and SUTN9-2 (**B**), respectively. Data is expressed as absorbance at 450 nm. Three and two positive clones showed specific binding to DOA9 and SUTN9-2, respectively, and not to 1% (v/v) BSA, which is used as a negative control.

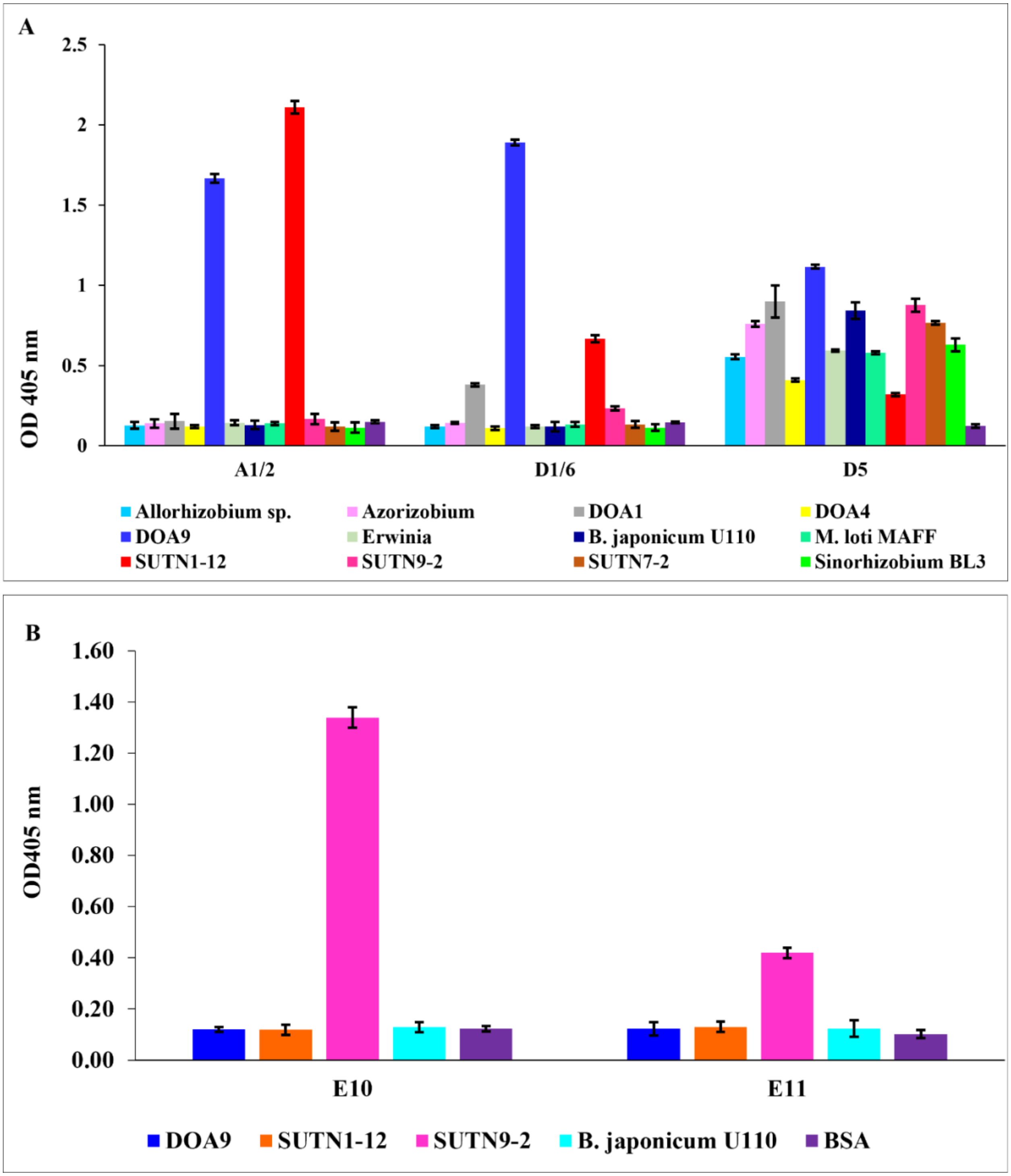

Figure S2. Specific binding of selected phage clones isolated from biopanning against *Bradyrhizobium* strain DOA9 (A) and SUTN9-2 (B). Different boiled bacterial strains were immobilized onto ELISA plate and tested for the binding against positive phage clones identified by phage ELISA (Fig. S1). One percent BSA was used as negative control. Values are the mean of triplicate wells. Error bars show the standard deviation for each set of data.

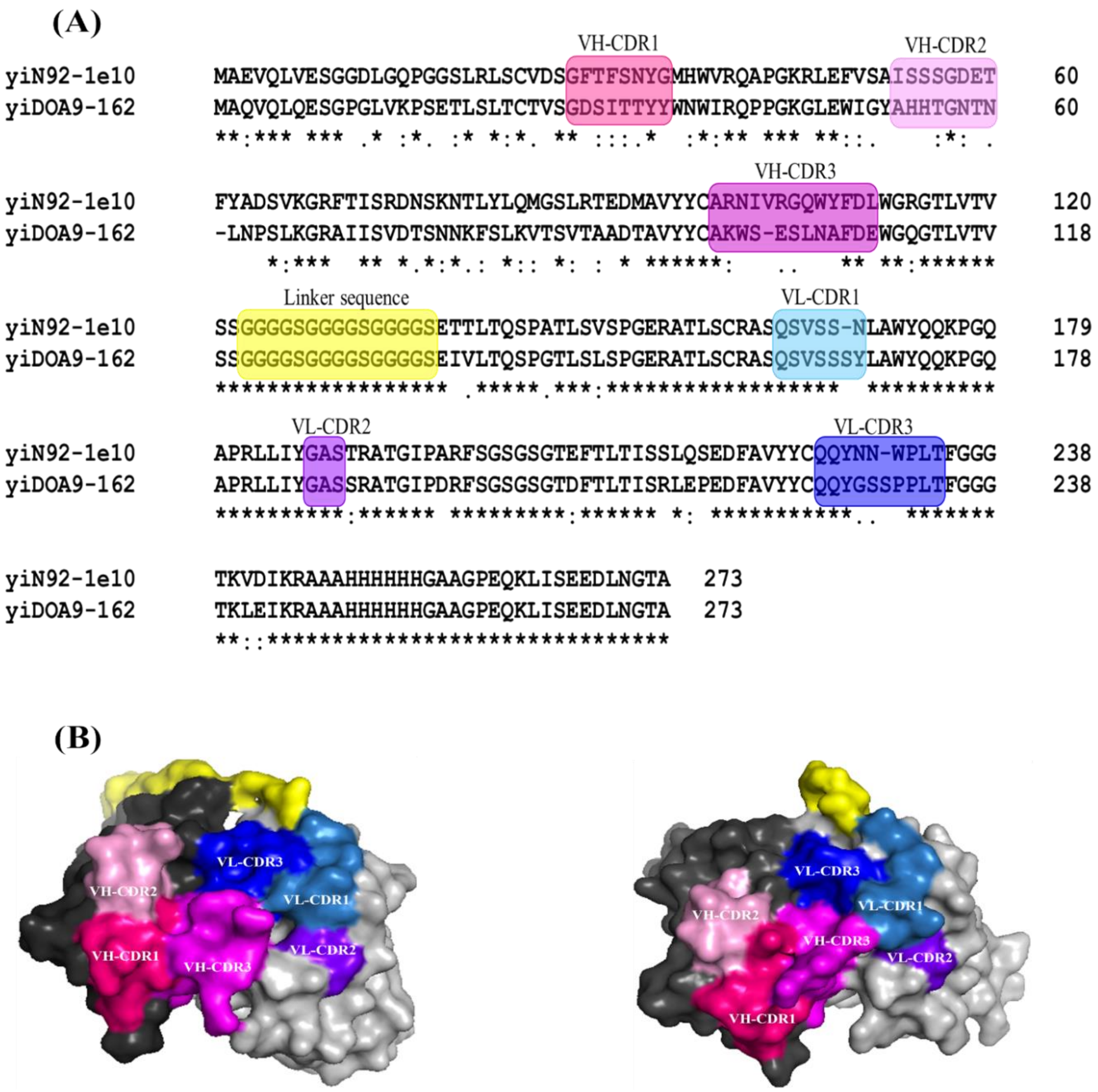

Figure S3. **Amino acid sequence analysis and three-dimensional structure prediction of selected scFv. (A)** The amino acid sequence of scFv antibody fragments of yiN92-1e10 and yiDOA9-162 are shown. The three complementary determining regions: CDR1, CDR2, CDR3 of VH are indicated in red-pink, pink and purple-pink color, respectively, GC-rich sequence that links VH and VL segments of these two scFv antibodies are indicated in yellow color, and CDR1, CDR2, CDR3 of VL are indicated in light-blue, purple-blue and blue color, respectively, **(B)** The predicted 3D structure of scFv yiN92-1e10 (left) and scFv yiDOA9-162 (right) are shown. Sequence alignment of the two scFv antibodies was done using Clustal Omega (https://www.ebi.ac.uk/Tools/msa/clustalo/).

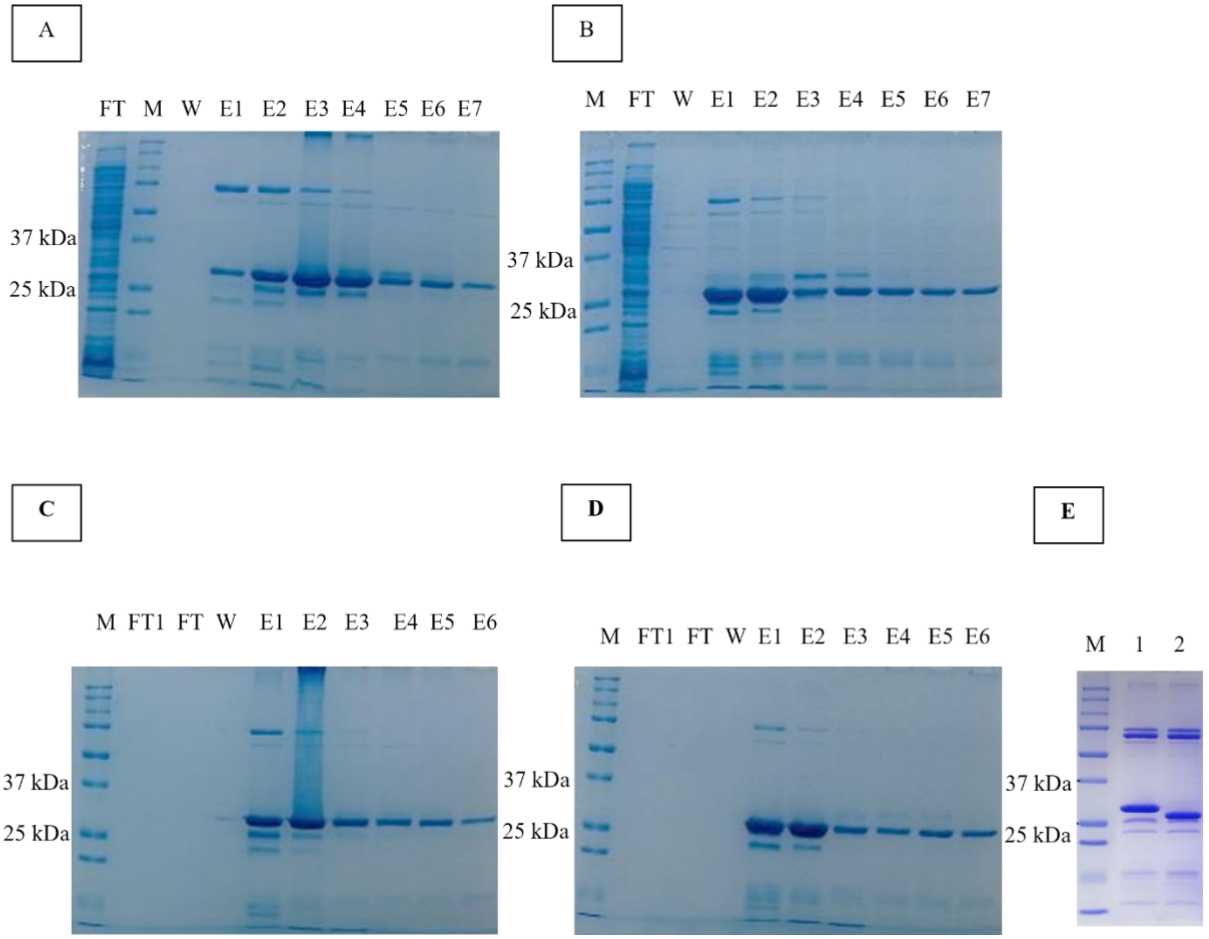

Figure S4. SDS-PAGE analysis of purified soluble scFv antibody. Upper panel illustrates the results from the first round of purification. Soluble scFv antibody against *Bradyrhizobium* strain DOA9 clone yiDOA9-162 **(A)**, and soluble scFv antibody against *Bradyrhizobium* strain SUTN9-2 clone yiN92-1e10 **(B)** were purified from cell lysate by IMAC. Lane M: protein molecular weight marker; lane FT: flow through fraction; lane W: wash fraction; lanes E1, E2, E3, E4, E5, E6, and E7 are the seven elution fractions. The soluble scFv antibody of approximately 30 kDa can be found in elution fractions E1 to E7. After the first round of purification, the eluted fractions from first purification were pooled together and purified again as illustrated in the bottom panel for clone yiDOA9-162 **(C)** and **(D)** yiN92-1e10. (**E**) SDS-PAGE illustrates protein bands of pooled eluted samples after the second round of purification. The soluble scFv antibody is approximately 30 kDa. Lane M: protein molecular weight marker; lane 1: purified scFv clone yiN92-1e10; lane 2: purified scFv clone yiDOA9-162.
